# Case Report: Embolization of a cognard type V cavernous sinus dural arteriovenous fistula via occluded Inferior petrosal sinus

**DOI:** 10.3389/fradi.2026.1795775

**Published:** 2026-04-10

**Authors:** Zhaolong Zhang, Yanan Jiang, Qi Zhang, Liming Shao, Hongzhang Xu, Yixing Xie, Xiaolong Zhao, Chengjian Sun, Rui Xu

**Affiliations:** 1 Department of Interventional Radiology, The Affiliated Hospital of Qingdao University, Qingdao, China; 2 Department of Anesthesiology, The Affiliated Hospital of Qingdao University, Qingdao, China; 3 Department of Neurosurgery, 411 Hospital of Shanghai University, Shanghai, China; 4 Department of Radiology, Weihai Municipal Hospital, Shandong Province, Weihai, China

**Keywords:** cavernous sinus, Cognard type V, dural arteriovenous fistula, occluded inferior petrosal sinus, Onyx embolization

## Abstract

**Background:**

Cavernous sinus dural arteriovenous fistula (CS-DAVF) with cortical venous drainage to the spinal perimedullary veins (Cognard type V) is a rare clinical entity. The treatment becomes particularly challenging when such a case is complicated by occlusion of the inferior petrosal sinus. We report a rare case of Cognard type V cavernous sinus dural arteriovenous fistula (CS-DAVF) embolized with Onyx via the occluded inferior petrosal sinus (IPS).

**Case description:**

A 62-year-old woman presented to our hospital with nausea, vomiting and progressive weakness in both lower limbs. Magnetic resonance imaging (MRI) revealed extensive edema of pons and medulla oblongata with flow voids. Cerebral angiography demenstrated a dural arteriovenous fistula (DAVF) in the left cavernous sinus, supplied by the meningohypophyseal trunk and branches of the middle meningeal artery. The fistula drained into the intercavernous sinus and right cavernous sinus, ultimately draining into the spinal perimedullary veins. Transarterial approach was tried first. Onyx-18 was injected into the branches of middle meningeal artery, partially casting the fistula. However, the DAVF was not completely occluded due to residual supply from the meningohypophyseal trunk. The second operation was performed using transvenous approach. The microcatheter was successfully navigated to the fistulous point via the occluded right IPS and the intercavernous sinus. Left cavernous sinus (CS) and intercavernous sinus were casted using Onyx-18 and the DAVF was completely eliminated.

**Conclusion:**

We report a Cognard type V CS-DAVF that was embolized with Onyx through an occluded IPS, demonstrating that this technique is a feasible and effective treatment option.

## 1 Introduction

Dural arteriovenous fistula (DAVF) is a lesion with an arteriovenous shunt in the dural membrane. The symptoms usually depend on the location and drainage pattern. Symptoms of patients with DAVF in the cavernous sinus (CS) usually are related to the eyes. The Cognard type V DAVF, which is drained into the spinal vein, is a rare subtype. Herein, we report a rare case of Cognard type V cavernous sinus dural arteriovenous fistula (CS-DAVF) embolized with Onyx via the occluded inferior petrosal sinus (IPS).

## 2 Case description

### 2.1 Medical history

**Figure 1:**
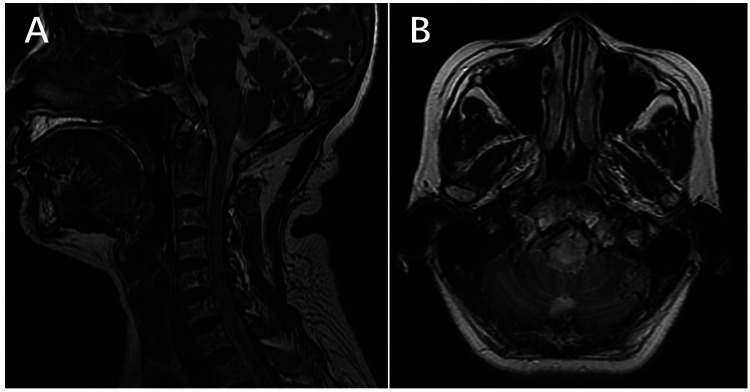
Preoperative magnetic resonance imaging (MRI). **(A)** and **(B)**: MRI showing extensive edema of pons with flow voids over its surface.

**Figure 2:**
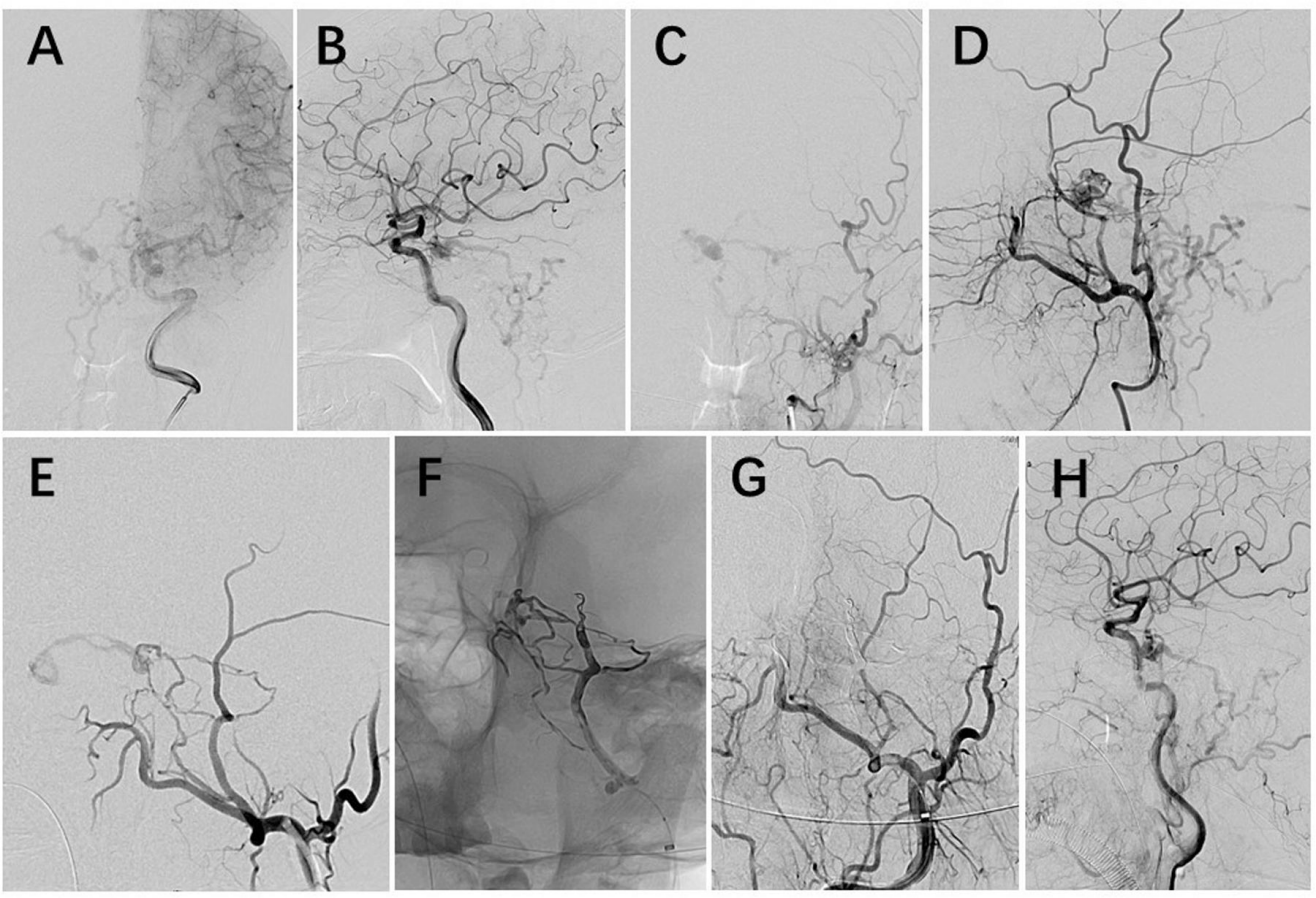
Angiography before the operation. **(A)** and **(B)**: Left internal artery angiogram showing a DAVF on the left cavernous sinus supplied by the meningohypophyseal trunk and drained into the spinal perimedullary vein in continuation with intercavernous sinus and right cavernous sinus. **(C)** and **(D)**: Left external artery angiogram demonstrating the same fistula also supplied by branches of the middle meningeal artery. **(E)**: The left external artery angiogram showing branches of the middle meningeal artery feeding the fistula on the left cavernous sinus. **(F)**: Plain radiography showing the final Onyx cast, mainly in the branches of middle meningeal artery and partially in the fistula. One coil was filled to block the distal middle meningeal artery, and Onyx-18 was injected using pressure cooker technique. **(G)**: Injection of left external artery showing the fistula was embolized. **(H)**: Injection of left internal artery showing the remained fistula was supplied by meningohypophyseal trunk.

A 69-year-old woman was admitted to our hospital. Half a year ago, she started to feel nausea accompanied by vomiting, dizziness and numbness in the right limbs. Gastroscopy showed nothing special. Her symptoms persisted, and five months later she suffered weakness of both legs. She required some help, but was able to walk without assistance with a modified Rankin Scale (mRS) 3. MRI was performed, and T2-weighted image showed extensive edema of pons and medulla oblongata with flow voids (Figure 1). Considering the flow voids around the pons and medulla oblongata, cerebral digital subtraction angiography (DSA) was performed. And a DAVF of the left cavernous sinus was found, which was fed by the meningohypophyseal trunk and branches of the left middle meningeal artery and drained into the spinal perimedullary vein in continuation with right cavernous sinus and intercavernous sinus (Figures 2A–D).

### 2.2 Endovascular treatment

The endovascular embolization was performed under general anesthesia and with full heparinization. We used the right transfemoral access and a 6-French guiding catheter (Envoy DA, Cordis Corporation, Florida, USA) was placed in the left external carotid artery (ECA). We tried to navigate a microcatheter (Marathon, Medtronic, Minnesota, USA) into the branch of middle meningeal artery (MMA) as close as possible to the nidus, but failed. Instead, the pressure cooker technique was used. To avoid the Onyx (Ev3, Irvine, CA) infiltration into the distal vessel of MMA, two coils was detached in the distal trunk of MMA to create a plug. Then the microcatheter (Echelon-10, Ev3, Irvine, CA) was placed proximal to the coils in the MMA. Before Onyx injection, we used a 3.0mm*20 mm Gateway balloon (Stryker, Michigan, USA) in the internal maxillary artery to block the origin of MMA, in order to prevent the Onyx from refluxing. After that, Onyx-18 was injected slowly and we could see the Onyx filling the branches of MMA feeding the sinus and the nidus point. Considering the nidus was also fed by the meningohypophyseal trunk (MHT) of internal carotid artery (ICA), we stopped the injection when the Onyx reached the nidus to avoid its reflux into ICA. Injection of ECA showed the occlusion of the DAVF, however the feeding of MHT remained (Figures 2E–H).

**Figure 3:**
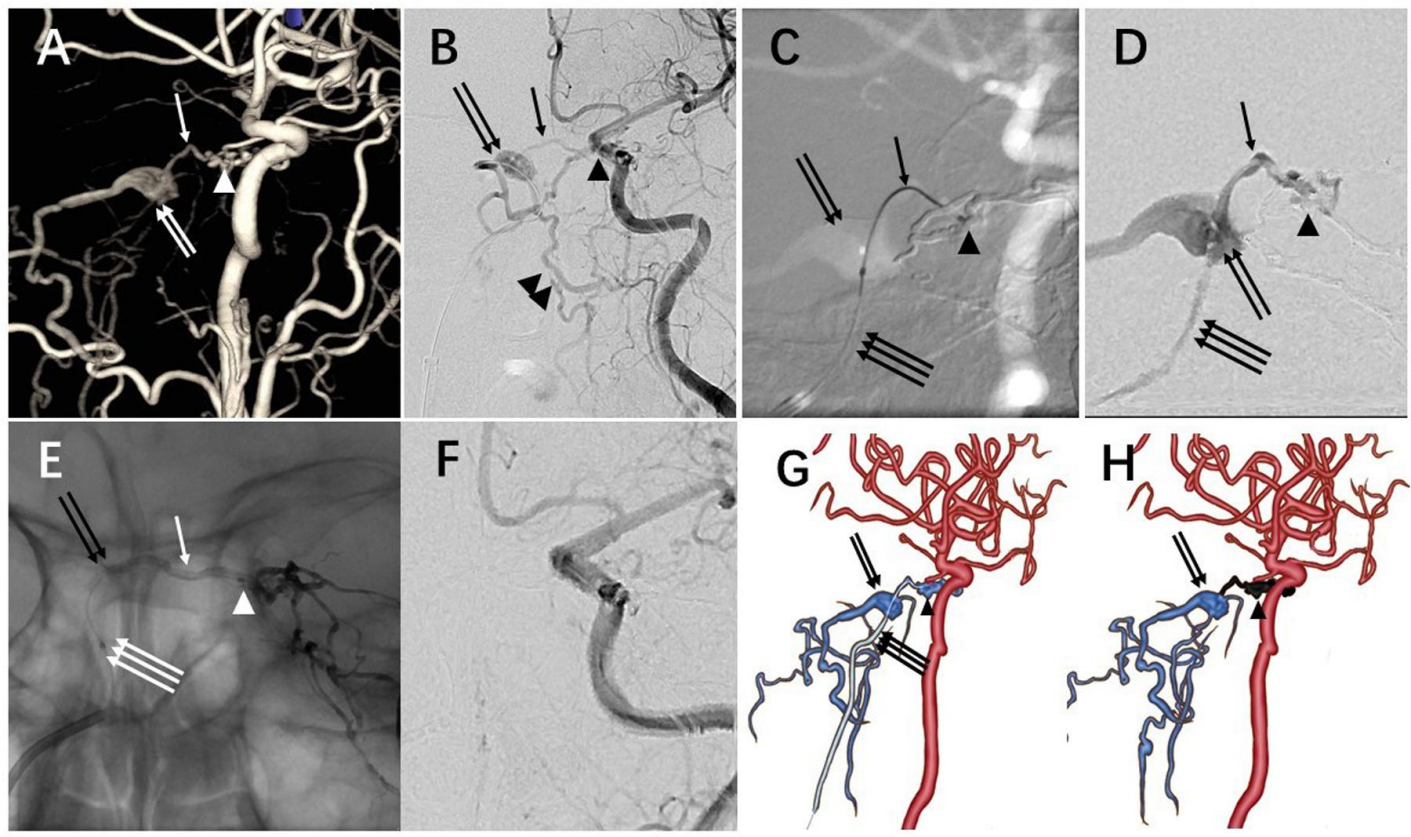
Second operation using transvenous approach. **(A)**: Three-dimensional DSA showing the fistula supplied by meningohypophyseal trunk. **(B)**: Access via the occluded right IPS was attempted by transvenous approach, and the angiography of left ICA verified the catheter introduced into the right CS successfully. **(C)**: The Echelon-10 (Ev3, Irvine, CA) microcatheter was navigated into the left CS via intercavernous sinus under roadmap. **(D)**: Microcatheter tip-injection showing the fistulous point and draining veins. **(E)**: Onyx-18 was injected under blank roadmap guidance, and Onyx-18 occluded the left CS fistulous site and intercavernous sinus. **(F)**: Injection of left ICA showing the fistula completely occluded. G: Schematic diagram of the DAVF and operation. Microcatheter was successfully navigated into the fistulous point. **(H)**: Onyx cast in the left CS and intercavernous sinus. Arrowhead: left CS fistula; Arrow: intercavernous sinus; Double arrow: right cavernous sinus; Triple arrow: right IPS; Double arrowhead: spinal perimedullary vein.

After the first operation, her symptoms were alleviated slightly. One month later, the second operation was performed. We attempted the venous access through the right femoral vein. A 4F diagnostic catheter with a continuous flush of heparin was placed in the left ICA to demonstrate the veins of DAVF. A 6F long sheath was advanced transvenously via the right internal jugular vein, and a 5F guiding catheter was then further introduced. Once the 5F catheter is advanced to the proximal aspect of the sigmoid sinus, it is rotated anteriorly and medially to access the anterior condylar confluence. After reaching the anterior condylar confluence, the 5F catheter is stabilized, and a 0.035-inch guidewire is carefully advanced in a medial, posterior, and superior direction, which allows it to traverse toward the inferior petrosal sinus. The 5F catheter is then advanced over the guidewire, and its position is confirmed by contrast injection to verify that the catheter has successfully entered the occluded inferior petrosal sinus. Inside the 5F guiding, a microcatheter (Echelon-10, Ev3, Irvine, CA) was navigated into the right cavernous sinus with the assistance of a microguidewire (Synchro 2, Stryker, Michigan, USA) (Figure 3B). Roadmap was performed using the 4F diagnostic catheter in the left ICA. The Echelon-10 (Ev3, Irvine, CA) microcatheter was advanced into the left cavernous sinus via intercavernous sinus under this roadmap. After the injection of the microcatheter confirmed that the microcatheter was located at the fistula sinus, Onxy-18 was injected slowly (Figure 3E). After the left cavernous sinus and intercavernous sinus were filled with Onyx, injection of the left ICA showed the DAVF was completely embolized with the left ICA patent (Figure 3).

### 2.3 Outcome

No periprocedural complication occurred for this patient. She was treated with low-molecular-weight heparin for 3 days before discharge and with warfarin for 1 month. Her symptoms were also relieved at 1 month follow-up. And at 2 year clinical follow-up, she achieved a good outcome without any neurological deficit and had a modified Rankin Scale of 0.

## 3 Discussion

CS-DAVF is the abnormal communication between the CS and the dural arteries from the internal carotid artery (ICA) and external carotid artery (ECA), which is commonly caused by CS recanalization after thrombosis (1). The symptoms of DAVF are related to location and the venous drainage pattern. The most common symptoms of CS-DAVF are ocular, namely proptosis and chemosis, since symptomatic CS-DAVF is usually drained to superior ophthalmic vein. However, the cs-DAVF case we reported suffered infractory nausea, vomiting and aggravated weakness of both lower limbs. Similar cavernous sinus dural arteriovenous fistulas have been documented previously (2, 3).Dural arteriovenous fistulas presenting with myelopathy due to spinal venous congestion are primarily located in the posterior cranial fossa. These include fistulas at the petrous apex and the craniocervical junction, among others. However, fistulas with the actual fistula site located within the cavernous sinus are relatively uncommon (4). When a dural arteriovenous fistula (DAVF) in the cavernous sinus region presents with myelopathic symptoms, the underlying mechanism is typically the obstruction of its normal drainage pathways, leading to eventual rerouting of venous flow toward the pontine and spinal veins. In this specific case, the fistula drained posteriorly via the right cavernous sinus into the superior petrosal sinus. Furthermore, occlusion was present just before the junction where the superior petrosal sinus meets the sigmoid sinus. This occlusion caused reflux into the transverse pontine vein, which would normally drain into the superior petrosal sinus. Consequently, the venous flow was redirected anteriorly to the pons and downward toward the spinal venous system.

Based on the morphology of the DAVF venous drainage, the two most commonly used classifications are the Borden classification and the Cognard classification (5). Type V of Cognard classification means the fistula has spinal perimedullary venous drainage, associated with progressive myelopathy. The DSA of the case we reported demonstrated that the DAVF was supplied by the meningohypophyseal trunk and branches of ECA and drained into the spinal perimedullary vein in continuation with cavernous sinus. This makes the case quite rare and only several cases have ever been reported (2, 3, 6). In one reported case, the dural arteriovenous fistula underwent spontaneous thrombosis during the procedure of superselective transvenous access (2). In another reported case, employing a hybrid approach with direct puncture of the cavernous sinus led to a successful cure of the lesion (3). There are also a case reported in the literature where a direct surgical approach via open craniotomy was employed to perform embolization of the cavernous sinus (7). A recent literature report described a case of a cavernous sinus dural arteriovenous fistula, Cognard type V, with drainage into the posterior circulation brainstem/spinal veins. Successful embolization was achieved via a trans-inferior petrosal sinus approach. Notably, in this particular case, the inferior petrosal sinus was patent prior to the procedure (8). We reviewed the literature on cavernous sinus dural arteriovenous fistulas with retrograde venous drainage to posterior fossa cortical veins leading to brainstem edema, and summarized the findings in Table 1. In particular, a recent publication described a case similar to ours—a cavernous sinus dural arteriovenous fistula with an occluded inferior petrosal sinus, which was embolized through occlude IPS using coils (9). However, our case was embolized using Onyx. So, to our best knowledge, this is the first case of Cognard type V CS-DAVF was embolized with Onyx via the occluded IPS.

**Table 1:** Summary of reported cases of cavernous sinus arteriovenous fistula with brain stem congestion.

Authors	Year	Age/Gender	Fistula location	Arterial feeders	Venous drainage pattern	Treatment/Access route	IPS	Embolic material	Angiographic/Clinical Outcomes
Uchino et al. (15)	1997	64 F	Left side	Bilateral ECA and left ICA	Basal vein of Rosenthal → Vein of Labbe	TAE	Occluded	PVA	Ongoing symptoms
Uchino et al. (15)	1997	72 M	Bilateral side	Bilateral ICA and ECA	Cortical veins of posterior fossa	TAE	Occluded	PVA	Improved
Takahashi et al. (6)	1999	49 M	Left side	Bilateral ICA	Left SOV, IOV, SPS and cortical veins of posterior fossa	TVE, left SOV	Occluded	Coils	Cured
Kai et al. (7)	2004	56 F	Right side	Right ICA and ECA	Petrosal vein → lateral mesencephalic vein	Surgery	Occluded	NA	Ongoing symptoms
Kai et al. (7)	2004	70 F	Right side	Bilateral ICA and ECA	Deep Sylvian vein → pontomesencephalic veins	Surgery	Occluded	NA	Improved
Iwasaki et al. (16)	2006	71 F	Right side	Right ICA and ECA	SPS → straight sinus, right cerebellar cortical veins and the inferior vermian vein	Radiation	Occluded	NA	Improved
Ko et al. (17)	2009	54 M	Right side	Right ICA and ECA	Pontomesencephalic veins	TAE and Radiation	Occluded	NBCA	Ongoing symptoms
Miyagishima et al. (18)	2012	80 F	Right side	Bilateral ICA and ECA	SOV, SPS, Petrosal vein	TAE and Radiation	Occluded	PVA	Improved
Muram et al. (3)	2017	70 F	Right side	Bilateral ICA and right ECA	Leptomeningeal vein of cerebellar hemisphere	Combined surgical and endovascular approach	Occluded	Coils	Cured and improved
Iampreechakul et al. (2)	2019	39 F	Left side	Bilateral ICA and left ECA	SPS, petrosal vein, lateral medullary vein and spinal vein	TVE	Occluded	Spontaneous Closure	Improved
Nambu et al. (8)	2020	77 F	Bilateral side	Bilateral ICA and ECA	Bilateral SOV, IPS, SPS and leptomeningeal vein of brainstem	TVE	Patent	Coils	Cured and improved
Goto et al. (9)	2024	73 M	Left side	Bilateral ICA	Spinal perimedullary veins	TVE	Occluded	Coils	Cured and improved
Present case	2026	62 F	Left side	Left ICA and ECA	Spinal perimedullary veins	TVE	Occluded	Onyx	Cured and improved

SPS, superior petrosal vein; SOV, superior ophthalmic vein; IOV, inferior ophthalmic vein; TAE, transarterial embolization; TVE, transvenous embolization; NA, not available.

The transvenous endovascular approach is the first option in treatment of most DAVFs now. As to CS-DAVF, it could be cured endovascularly via the IPS, superior ophthalmic vein, superpetrosal sinus, pterygoid plexus and other veins (10–12). The IPS approach is the safest, shortest and simplest route to the cavernous sinus and should be chosen first. When the IPS is nonvisualized during angiography, it does not exclude the possibility of successful catheter navigation to the cavernous sinus (13). Transvenous embolization via recanalization of occluded sinuses is now more frequently successful and safe, a progress driven by the deeper anatomical understanding of these fistulas (14). For this patient, our initial attempt via an arterial approach failed to achieve complete occlusion of the fistula. Consequently, a transvenous approach was adopted during the second-stage procedure. Following this case, our center has since prioritized the strategy of recanalizing the occluded inferior petrosal sinus as the primary treatment for cavernous sinus dural arteriovenous fistulas presenting with inferior petrosal sinus occlusion. Based on our experience, during the transvenous recanalization procedure, a 0.035-inch hydrophilic guidewire guided with a single-curve angiographic catheter tends to preferentially enter the anterior condylar confluence. Performing an angiogram at this location often reveals subtle clues indicating the course of the occluded sinus. If the pathway remains uncertain, performing a 3D rotational angiogram using the catheter positioned within the anterior condylar confluence can frequently delineate the trajectory of the inferior petrosal sinus, thereby guiding successful recanalization.

Regarding the selection of the approach whether to recanalize the left or right inferior petrosal sinus for this particular patient, the following considerations were pivotal. Although the fistula was located in the left cavernous sinus, the opacified portion of the left sinus was small, suggesting the presence of multiple compartments. Even if the left inferior petrosal sinus were successfully recanalized, reaching the actual fistula site might have been difficult. In contrast, the fistula drained via the intercavernous sinus into the right cavernous sinus, which demonstrated a larger opacified volume. Therefore, recanalizing the right inferior petrosal sinus offered a higher probability of accessing the relevant drainage compartment and, consequently, the fistula itself. Based on this rationale, we prioritized the right-sided trans-inferior petrosal sinus approach. The procedure proceeded as anticipated, with the microcatheter successfully reaching the fistula nidus. Complete occlusion was achieved using a minimal amount of Onyx liquid embolic agent, resulting in an excellent clinical and angiographic outcome.

## 4 Conclusion

The authors reported a quite rare case of Cognard type V CS-DAVF, which was completely embolized with Onyx via the occluded IPS. It is a feasible alternative access route for transvenous embolization of CS-DAVF, even when the IPS is occluded.

## Data Availability

The original contributions presented in the study are included in the article/Supplementary Material, further inquiries can be directed to the corresponding author/s.
